# The Impact of a Multidisciplinary Approach Protocol and Integrated Guidelines for Antibiotic Prophylaxis in Plastic Surgery Procedures

**DOI:** 10.29252/wjps.10.3.54

**Published:** 2021-09

**Authors:** Isa AlAlwani, Hasan AlTahoo, Fatima Yaqoob, Fatema Ahmed Ali, Sadeq Alekri

**Affiliations:** 1 Department of Plastic Surgery, Salmaniya Medical Complex, Manama, Kingdom of Bahrain

**Keywords:** Plastic surgery, Surgical site infections, Wound infections, Guidelines, Protocol, Aesthetic, Antibiotic prophylaxis

## Abstract

**BACKGROUND:**

Surgical antibiotic prophylaxis has been widely used for prevention of surgical site infections (SSI’s). World Health Organization (WHO) global guidelines strongly recommend the administration of pre-operative prophylactic antibiotic, depending on the type of surgery, to reduce SSI’s. However, within Gulf Cooperation Council (GCC) countries, antibiotic resistance has been rising due to unregulated prescribing practice. We aimed to assess adherence to local/international guidelines in the plastic surgery unit of Salmaniya Medical Complex.

**METHODS:**

This study was a retrospective review of adults’ undergoing plastic surgery between the dates of 1^st^ of January 2019 to 30^th^ of April 2019. Recommendations and guidelines were provided by South Australian Guidelines for Surgical Antimicrobial Prophylaxis, NHS Greater-Glasgow Foundation Trust. Salmaniya Medical Complex Guidelines were also taken into consideration. This was followed by an implementation of standardized guidelines and a re-assessment period for another four months.

**RESULTS:**

There were 106 patients who met the inclusion/exclusion criteria throughout the primary cohort. With respect to choice and dose of antibiotics, only 21 (19.8%) of the procedures were adherent to global/local guidelines. Similarly, only 11.5% of those cases have met the recommended timing for pre-operative antibiotic administration. After the implementation period, adherence to guidelines regarding choice and time of antibiotic administration has increased to 36.8% and 32.6% respectively. SSI decreased from 1.8% to 0.08%.

**CONCLUSION:**

Practice in SMC in plastic surgery pre-operative antibiotic prophylaxis shows poor compliance to both local and international guidelines in terms of choice, dose, and time of administration. We were able to significantly improve adherence to international/local practice in both areas by implementing an integrated protocol in liaison with the medical staff involved in the plastic surgery unit and operating theatres.

## INTRODUCTION

Surgical Site Infection (SSI) refers to an infection that takes place post-operatively involving the part of the body where the surgery took place. SSI is a main factor that increases hospitalization being the third most frequent cause of nosocomial infection and affecting 16% hospitalized in-patients. In surgical patients, SSIs accounted for 77% of deaths due to post-operative complications^1^. As one of the most important medical measures to reduce SSIs, the WHO global guidelines strongly recommend the administration of pre-operative prophylactic antibiotic depending on the type of surgery^2^.

Within the literature, the effectiveness of pre-operative antibiotics in preventing post-operative infections in plastic surgery operations has been widely demonstrated ^3^^-^^6^. One particular study has illustrated that in clean contaminated procedures, prophylactic antibiotics prevents wound dehiscence, bone mal-union, stitch and septal abscesses^7^. One prospective observational study shows that the incidence of postoperative infections in non-antibiotic groups reach up to 42% compared to 8.9% in antibiotic groups^3^. 

Despite that, there is a growing antibiotic crisis with new strains of drug resistant bacteria encountered in wounds^8^. Within Gulf Cooperation Council (GCC) countries, antibiotic resistance has been rising compared to European countries, due to unregulated prescribing practice and unaudited use of antibiotics^9^. As a result, there is a definite need to regulate the use of prophylactic antibiotics through implementing local/international guidelines. 

In plastic surgery surgical prophylaxis, there is a customization of guidelines in different countries and hospitals based on local resistance patterns and types of pathogens. However, there are some common relations between most guidelines. Many guidelines, like the National Health Service (NHS) and South Australia guidelines, agree on the need of antibiotic prophylaxis on clean-contaminated, contaminated and dirty procedures ^10^^-^^12^. In addition, there is evidence that antibiotics are not recommended for clean procedures ^10^^-^^13^.

We aimed to evaluate the adherence and accuracy of giving peri-operative antibiotic prophylaxis in plastic surgery with respect to local/global guidelines, in the largest hospital in the Kingdom of Bahrain, Salmaniya Medical Complex. 

## METHODS

*Design and standards*

This study was a retrospective, standards based clinical review, evaluating peri-operative antibiotics given to all the patients who underwent operations in the plastic surgery unit in Salmaniyya Medical Complex between the 1^st^ of January 2019 and the 30^th^ of April 2019. The local guidelines on surgical antibiotic prophylaxis by Salmaniya Medical Complex was used as the main standard in this study^14^. Recommendations provided by NHS Salisbury^15^, NHS Greater Glasgow Foundation Trust guidelines^16^, and South Australian guidelines for Surgical Antimicrobial Prophylaxis^17^ were adopted where the local guidelines were insufficient.

*Study settings and inclusion and exclusion criteria*

All adult and pediatric cases (of 1 year or more), who underwent plastic surgery operations under general anesthesia, over the four-month period between the 1^st^ of January 2019 and the 30^th^ of April 2019 were reviewed. Guidelines were implemented after the initial assessment period and were re-assessed between the dates of 1^st^ September 2019 to 31^st^ December 2019. Patients who underwent surgery under local anesthesia, received antibiotics as treatment, had incomplete data; were excluded from the study. 

*Data collection and analysis*

The data collection included three main categories. The first category was patient demographics and medical background. This included patient identification, age, ethnicity, weight (in kilograms), known allergies and history of adverse reactions to antibiotics, colonization with resistant organisms and chronic comorbidities. The second category was operation details; where details of the operation performed, classification, type of anesthesia used, name of operating surgeon, time of the surgical incision and duration of the operation were noted. The third category was the choice of antibiotic prophylaxis (peri-operative and post-operative) administered including the name, dose, time of administration and reason for administering/withholding a particular drug as antibiotic prophylaxis.

Data from electronic and written hospital files were directly plotted into an Excel spreadsheet by the authors. National ID numbers of cases in the inclusion criteria were recorded from the plastic surgery department and were used to access patient files. Data analysis was completed through Excel (2016 version).

*Implementation period and re-assessment*

Results obtained during the first study period were used to design a quality improvement strategy. An implementation period of three months was given, after which, a retrospective re-assessment was conducted using the same methodology from 1^st^ September to 31^st^ December 2019. 

## RESULTS

The study took place over a period of one year, which included a three-month period for quality improvement strategies implementation. A total of 236 patients were admitted to the Plastic Surgery Department in Salmaniyya Medical Complex. Out of these cases, 112 had undergone operations/procedures under general anesthesia and received prophylactic antibiotics. After removal of patients with incomplete documented data, a total of 106 patients were included in the primary study. During the 3 months from Sep to Dec 2019, 117 patients met the criteria and were included in the re-assessment period. 

*Operation Classification*

Within plastic surgery, operations are generally classified into four types: clean, clean-contaminated, contaminated, and dirty. For the purpose of this study, clean procedures have been further divided into aesthetic (abdominoplasties, liposuction, breast surgeries, and implant insertions), uninfected burns (with or without skin grafts), and others (no involvement of implants, grafting, or open fractures). The procedures analyzed during the study period are described in table one (Table 1). 

*Choice of prophylactic peri-operative antibiotics*

In all operations accounted for in the primary study, only 19.8% of the cases were given appropriate antibiotic prophylaxis consistent with local and international guidelines (Table 2). In specific: clean, clean-contaminated and dirty operations were given prophylaxis appropriately in 15.5%, 0%, and 100% respectively. In comparison, within the re-assessment study clean, clean-contaminated and dirty procedures had adherence of 31.8%, 0% and 100% respectively. Intravenous route was used in administering peri-operative antibiotics in all operations (Table 3).

*Clean operations*

In peri-operative prophylaxis of aesthetic procedures, our local hospital guidelines encourage a single dose of cefazolin 1g IV as a STAT dose. Out of 69 patients undergoing aesthetic surgeries in the primary study, 65 patients were prescribed antibiotics pre-operatively as required. However, the antibiotics prescribed in all cases were not the antibiotic of choice. All cases were prescribed either ceftriaxone (third generation antibiotic) or cefuroxime (second generation antibiotic) (Table 4). Additionally, it is noteworthy that all cases had been prescribed an additional course of oral cefuroxime post-operatively, which is not in accordance with current best practice. With regards to uninfected burns, 7 cases (70%) were not prescribed any prophylaxis. On the contrary, only three cases where given prophylactic peri-operative antibiotics (Table 4 and 5). Yet, the prophylactic antibiotic was not the antibiotic of choice (Table 4). Other clean procedures that do not require peri-operative prophylaxis, under local and NHS guidelines, include excision of skin lesions/keloids/cysts, cut wounds and hand surgeries. Within the primary study, 28 clean procedures fit this category, 14 of which were treated with adherence to guidelines by not being prescribed prophylactic antibiotics. On the contrary, the other four procedures were prescribed antibiotics unnecessarily. 

In aesthetic operations and uncomplicated burns, our facility’s practice was not adherent to current practice. On the contrary, other simple clean procedures had adherence of 83% to guidelines during the primary study (Table 5). During the re-assessment period, aesthetic procedures, uninfected burns and other clean procedures had an adherence of 23%, 63% and 59% respectively. Any operation where choice of antibiotic prophylaxis was not adherent to guidelines, the dose of antibiotic was not assessed (Table 5).

*Clean-contaminated and Dirty Procedures*

 With regards to clean-contaminated procedures, our facility was not adherent to guidelines during the study period. Three cases of cleft lip/cleft palate were considered within this category; all of given cefuroxime (Table 6). All cases classified as dirty (6 during primary study and 9 during re-assessment), were adherent to international and local guidelines, as all were treated with antibiotics as therapy depending on culture and sensitivity (Table 6 and 7).

*Time of administration of prophylactic antibiotics*

**Fig. 1:**
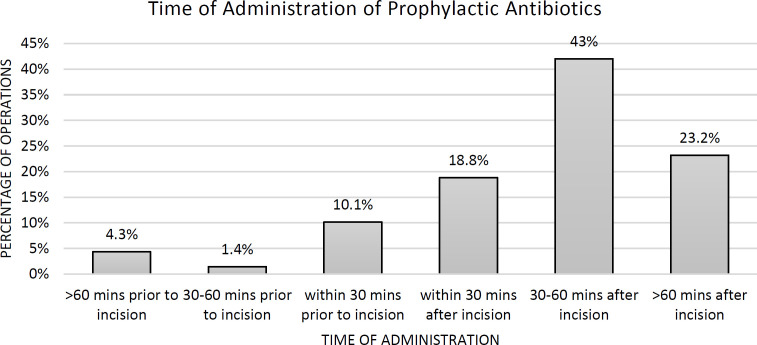
Timing of prophylactic antibiotic administration as observed during the primary study

It is recommended that IV peri-operative antibiotics are to be administered within 1 hour prior to surgical incision according to local hospital guidelines. Within the plastic surgery department in our treatment facility, there was significant non-compliance with regards to this element. Only 11.5% of operations were adherent to timely administration of antibiotics (Figure 1). The NHS guidelines further recommend that antibiotic prophylaxis is most effective within 30 min prior to incision, and only 10.1% of cases meet this recommendation (Figure 1). It is also noteworthy that in 85% of operations, prophylactic antibiotics were administered after incision, most of given 30-60 min after incision (43%).

*Re-assessment Period*

Following the re-assessment, we observed a significant improvement, with a 36.8% adherence to the recommendations in total procedures, in comparison with 19.8% total adherence within the primary study (Table 2). Furthermore, there was an increase in adherence to current best practice in our re-assessment by 23% in aesthetic procedures and 63% in uninfected burns. We observed a decrease of 19% adherence in prophylactic antibiotic use within the “other clean procedures” category. Dirty operations observed during the re-assessment had a compliance of 100% (Table 2). With regards to timely administration of prophylactic antibiotics, 32.6% of procedures during the re-assessment have met the required standards in comparison to 11.5% in the primary study (Table 2). The effect of this intervention has been reflected in the decrease of SSI’s from 1.8% in the primary study to 0.08% in the re-assessment study. 

## DISCUSSION

The prescribing practice of prophylactic antibiotics within plastic surgery has been an area of controversy, due to the lack of randomized controlled trials examining the role of prophylactic antibiotics in plastic surgery. Evidence has supported the use of prophylactic antibiotics in various contaminated surgeries and clean breast surgeries^18^^-^^29^. Even though abdominoplasties are classified as clean procedures, the use of prophylactic antibiotics is recommended within most guidelines, taking into consideration the prolonged operation time, extensive dissection and significant dead space^15^_._

We observed a pattern of over-prescribing in elective aesthetic surgeries, including the use of broad-spectrum antibiotics and prescribing a course of antibiotics post-operatively, which could be a result of common practice and surgeon preference to reduce a feared risk of complications in an elective surgery. Similar practice was also noted in a cross-sectional study, surveying surgeons on their prophylactic antibiotic use^30^. In clean aesthetic operations, cefazolin 1g IV stat is recommended, yet our hospital had limited supply of the drug. On the other hand, in the majority of the surgeries involving uninfected burns not covered by prophylactic antibiotics, increasing the chances of surgical site infections. 

Within our hospital, most antibiotic use that was not adherent to the recommendations was due to the inappropriate choice of the antibiotic being used. For example, according to current best practice, it is recommended that co-amoxiclav for prophylaxis is used for cleft lip/palate procedures.^17^ However, our facility used cefuroxime in most of these operations.

More to the point, we observed a preferential tendency towards the use of broad-spectrum antibiotics, especially third generation cephalosporins. Global Point Prevalence Survey of Antimicrobial Consumption and Resistance (Global-PPS) in 2017 revealed high rates of use of cephalosporins in general at our hospital and third generation cephalosporins in the surgery department in particular, which explain the significantly high use of third generation cephalosporin within our studied population^30^. 

The excessively inappropriate use of antibiotics has been a major cause of resistant bacterial strains and *Clostridium* *difficile* infections, causing a burden on both the patient and the healthcare system^31^.

Global Antimicrobial Resistance and Use Surveillance System (GLASS) Report published in 2020 by the WHO showed that 64.5% of patients from Bahrain who had *Staphylococcus aureus* bacteria isolated from blood samples, had isolates susceptible to oxacillin (a narrow- spectrum b-lactam). This suggests that the use of narrow-spectrum beta-lactams such as the recommended flucloxacillin would still be effective in the majority of cases. It was also reported that resistance pattern to cephalosporin included 50% of E. coli and 45% of K. pneumoniae. Unfortunately, insufficient data was available to calculate the Drug Resistance Index of cephalosporin in Bahrain^18^. Resistance and sensitivity pattern in the local population needs to be studied for better antibiotic use guidance. It is well-known that the recommended administration of the antibiotic is prior to incision, in order to obtain the required level of antibiotic at the site of surgery^19^. The majority of cases at our hospital received prophylactic antibiotics after the incision was made, creating a risk of surgical site infection. 

*Quality improvement strategies*

After conducting the primary study, we studied the areas of improvement in our practice. The results of the study were revealed and discussed within the plastic surgery department in the form of a meeting and summary leaflets were distributed to all plastic surgeons for feedback. Weekly summaries of the practice were reviewed in the morning meeting at the end of each week during the implementation period, during which points of improvement and common mistakes were further reinforced. Theatre staff played a role in ensuring that prophylactic antibiotics were given during the recommended time pre-operatively. We included a section on timing of preoperative antibiotic prophylaxis, adopted from the WHO Surgical Safety Checklist, within our local preoperative checklist and the supply of cefazolin was discussed with the pharmacy department. 

We observed a deficiency in the recommendation details within our local guidelines in the following areas: burns needing surgical intervention, clean operations not involving grafts or open fractures and clean-contaminated procedures. The importance of adding the mentioned areas within our local guidelines were discussed with the head of the plastic surgery department. 

**Table 1:** Total number of operations and their classification included in the study

		Primary Study		Re-assessment	
Operation Classification		Number of operations	Total operations (%)	Number of operations	Total operations (%)
Clean	**Aesthetic**	69	65	70	60
	**Uncomplicated burns**	10	9	15	13
	**other**	18	17	22	19
Clean / contaminated		3	3	1	1
Contaminated		0	0	0	0
Dirty		6	6	9	8
Total		106	100	117	100

Total number of operations included throughout this study and their relevant operation classification. The operations have been expressed in numbers and percentage

**Table 2:** Adherence to guideline standards in the primary and re-assessment durations of the study

**Guideline Standards**	**Primary Study Results**		**Re-assessment Results**	
**Choice of antibiotics and dose**				
Clean Operations				
1.1 For aesthetic procedures including abdominoplasties, liposuction, breast surgeries, and implant insertions, all cases are to be given a single dose of prophylactic antibiotics.	65/69	94%	70/70	100%
1.2 For aesthetic procedures including abdominoplasties, liposuction, breast surgeries, and implant insertions: cefazolin 1g IV STAT is the prophylactic antibiotic of choice	0/65	0%	16/70	23%
1.3 For burns requiring any type of surgical intervention: all cases are to be given a single dose of prophylactic antibiotics.	4/10	40%	8/15	53%
1.4 For burns requiring any type of surgical intervention: single dose of flucloxacillin 1g IV pre-operatively	0/4	0%	5/8	63%
1.5 For clean operations that do not involve implants, grafting, or open fractures: no antibiotic prophylaxis is required	15/18	78%	13/22	59%
Total adherence in clean operations	15/97	15.5%	34/107	31.8%
Clean-contaminated Operations				
2.1 For clean-contaminated procedures all cases are to be given a single dose of prophylactic antibiotics	3/3	100%	1/1	100%
2.2 For clean-contaminated procedures involving cleft lip/palate in pediatric : all cases are to be given a single dose of co-amoxiclav 30mg/kg	0/3	0%	0/1	0%
Dirty Operations				
3.1 For dirty procedures antibiotics are to be given therapeutically depending on culture and sensitivity.	6/6	100%	9/9	100%
**TOTAL ADHERENCE TO GUIDELINES**	**21/106**	**19.8%**	**43/117**	**36.8%**
**Time of administration of prophylactic antibiotics**				
4.1 Prophylactic antibiotics should be administered within one hour prior to surgical incision. All antibiotic administration must be complete at time of surgical incision	**8/69**	**11.5%**	**31/95**	**32.6%**

Note: standards 1.1, 1.2, 3.1, and 4.1 were adapted from local hospital guidelines. Standards 1.3, 1.4, 1.5, 2.1, and 2.2 were adapted from the NHS guidelines on surgical prophylaxis.

**Table 3:** Level of compliance in the all classes of operations in primary study and re-assessment period

**Antibiotic prophylaxis - adherence to guidelines**								
	**Clean**			**Clean-contaminated**			**Dirty**	
	**Primary Study**	**Re-assessment**	**Primary Study**		**Re-assessment**	**Primary Study**		**Re-assessment**
**Total number of operations (** ***n)***	97	107	3		1	6		9
**Compliant antibiotic prophylaxis use (** ***n)***	15	34	0		0	6		9
**Non-compliant antibiotic prophylaxis use ** ***(n)***	82	73	3		1	0		0
**Adherence to guidelines (%)**	15.5%	31.8%	0%		0%	100%		100%

**Table 4:** Level of compliance to the recommended choice and dose of antibiotic prophylaxis in clean plastic operations

**Frequency and percentages of antibiotics used in clean operations**		
**Clean operations - aesthetic**		
Name and dose of antibiotic	Frequency *n *(in %) Primary study	Frequency *n *(in %) Re-assessment
Ceftriaxone	21 (32)	19 (27)
Cefuroxime 1.5g	31 (48)	22 (31)
Cefuroxime 1g	1 (1.5)	2 (3)
Cefuroxime 750mg	12 (18.5)	11 (16)
Cefazolin 1g (recommended)	0 (0)	16 (23)
**Clean operations – uncomplicated burns**		
Name and dose of antibiotic	Frequency *n *(in %) Primary study	Frequency *n *(in %) Re-assessment
No prophylaxis given	7 (70)	7 (47)
Cefuroxime 750mg	3 (30)	3 (20)
Flucloxacillin 1g (recommended)	0 (0)	5 (33)
**Clean operations – other clean procedures**		
Name and dose of antibiotic	Frequency *n *(in %) Primary study	Frequency *n *(in %) Re-assessment
Cefuroxime 1.5g	1 (5.6)	3 (33)
Cefuroxime 750mg	2 (11.1)	6 (67)
No prophylaxis given (recommended**)**	15 (83.3)	13 (59)

Choice and dose of antibiotic prophylaxis given in clean operations throughout the study in comparison to recommended choice and dose in local and international guidelines. Table 2 shows more details on guideline standards and compliance levels.

**Table 5:** Total level of compliance in clean plastic operations

**Clean Operations - antimicrobial practices in plastic surgery**						
**Antibiotic prophylaxis practice**	Aesthetics *n*(in%)		Burns *n*(in%)		Others *n*(in%)	
**Study Period**	Primary study	Re-assessment	Primary study	Re-assessment	Primary study	Re-assessment
**Total number of operations**	69	70	10	15	18	22
**Given antibiotics**	65/69 (94)	70 /70 (100)	3/10 (30)	8/15 (53)	3/18 (17)	9/22 (41)
**Not given antibiotics**	4 /69 (6)	0/70 (0)	7/10 (70)	7/15 (47)	15/18 (83)	13/22 (59)
**Correct dose**	n/a	16/70 (23)	n/a	5/8 (63)	n/a	n/a
**Adherence to guidelines ** ***n *** **(in %)**	0 /69 (0)	16/70 (23)	0 /10 (0)	5/15 (63)	15/18 (83)	13/22 (59)

Antibiotic prophylaxis compliance in clean plastic surgery operations to local and international guidelines. Table 2 shows more details on guideline standards and compliance levels.

**Table 6:** Total level of compliance in clean-contaminated and dirty operations

**Clean-contaminated and Dirty Operations - antimicrobial practices in plastic surgery**				
Antibiotic prophylaxis practice	Clean contaminated *n*(in%)		Dirty *n*(in%)	
	Primary Study	Re-assessment	Primary Study	Re-assessment
**Total number of operations**	3	1	6	9
**Given antibiotics**	3/3 (100)	1/1 (100)	6/6 (100)	9/9 (100)
**Not given antibiotics**	0/3 (0)	0/1 (0)	0/6 (0)	0/9
**Correct dose**	n/a	n/a	6/6 (100)	9/9 (100)
**Adherence to guidelines ** ***n *** **(in %)**	0/3 (0)	0/1 (0)	6/6 (100)	9/9 (100)

Antibiotic prophylaxis compliance in clean-contaminated and dirty plastic surgery operations to local and international guidelines. Table 2 shows more details on guideline standards and compliance levels.

**Table 7:** Level of compliance to the recommended choice and dose of antibiotic prophylaxis in clean-contaminated and dirty operations

Frequency and percentages of antibiotics used in selected operations		
**Clean-contaminated operations ** *n *(in%)		
Name and dose of antibiotic	Primary Study	Re-assessment
Cefuroxime 750mg	3 (100)	1 (100)
Co-amoxiclav (recommended)	0 (0)	0 (0)
**Dirty operations – uncomplicated burns** * n *(in%)		
Name and dose of antibiotic	Primary Study	Re-assessment
Co-amoxiclav 1.2g	2 (33)	4 (44)
Ceftriaxone 2g	1 (17)	2 (23)
Meropenem 1g	2 (33)	3 (33)
Vancomycin 1g + meropenem 1g tds	1 (17)	0 (0)

Choice and dose of antibiotic prophylaxis given in clean-contaminated and dirty operations throughout the study in comparison to recommended choice and dose in local and international guidelines. Table 2 shows more details on guideline standards and compliance levels.

## LIMITATIONS

The main limitation to our paper as a retrospective study in the initial phase was the quality of documentation and incomplete records, in some cases. The short duration of time had a significant impact on the number of procedures within each category, with some categories having very limited cases affecting the overall compliance result. A re-dose of an antibiotic was not considered as most of the operations in our unit and within this study, did not surpass the 3-hour window required an additional dose. Lastly, a larger sample can be used to produce a statistically significant result for future studies. 

## CONCLUSION

The main aim of the study was to identify areas of improvement within the current practice of preoperative antibiotic use in the plastic surgery department at our hospital. We achieved an increase of 17% and 21.1% in adherence to recommendations regarding choice and time of antibiotics given respectively. The SSI rate decreased from 1.8% to 0.08% after the protocol intervention. As a result, we contributed to the development of strategies that would aid in encouraging adherence to the guidelines within our department. The success of implementing our quality improvement strategies was aided by a multidisciplinary approach between plastic surgeons, microbiologists, anesthetists and nursing staff; this included opportunity for regular feedback on current practice and the implementation of the required areas in our local preoperative checklist and guidelines. 

## FINANCIAL DISCLOSURE

No sources of funds, products, substances, or devices were used in conducting this article. There are no financial or commercial relationships/associations related to any of the authors. 

## CONFLICT OF INTERESTS

The authors have no conflicts of interest to declare.
